# Molecular distinctions of bronchoalveolar and alveolar organoids under differentiation conditions

**DOI:** 10.14814/phy2.16057

**Published:** 2024-06-02

**Authors:** Yan Yu, Zexin Chen, Bin Zheng, Min Huang, Junlang Li, Gang Li

**Affiliations:** ^1^ Nanfang Hospital Southern Medical University Guangzhou China; ^2^ Guangdong Research Center of Organoid Engineering and Technology Guangzhou China; ^3^ Guangzhou No.3 High School Guangzhou China

**Keywords:** alveolar model, bronchoalveolar organoid model, molecular signature, multidimensional analysis, validation system

## Abstract

The bronchoalveolar organoid (BAO) model is increasingly acknowledged as an ex‐vivo platform that accurately emulates the structural and functional attributes of proximal airway tissue. The transition from bronchoalveolar progenitor cells to alveolar organoids is a common event during the generation of BAOs. However, there is a pressing need for comprehensive analysis to elucidate the molecular distinctions characterizing the pre‐differentiated and post‐differentiated states within BAO models. This study established a murine BAO model and subsequently triggered its differentiation. Thereafter, a suite of multidimensional analytical procedures was employed, including the morphological recognition and examination of organoids utilizing an established artificial intelligence (AI) image tracking system, quantification of cellular composition, proteomic profiling and immunoblots of selected proteins. Our investigation yielded a detailed evaluation of the morphologic, cellular, and molecular variances demarcating the pre‐ and post‐differentiation phases of the BAO model. We also identified of a potential molecular signature reflective of the observed morphological transformations. The integration of cutting‐edge AI‐driven image analysis with traditional cellular and molecular investigative methods has illuminated key features of this nascent model.

## Graphical abstract

Graphic abstract of work‐flow of this study.

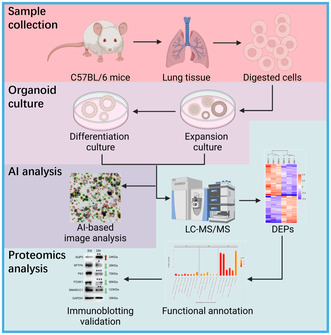

## 1 INTRODUCTION

Immortalized airway epithelial cell models and animal models have traditionally served as standard tools for studying the lung disease and biological process related to lung homeostasis (Hahn et al., 2002; Kemp et al., 2008; Kuehn et al., 2016). However, while cell line models lack the spatial structure, and animal models pose challenges due to cross‐species differences and ethical concerns regarding animal welfare (Anjum et al., 2022; Cioccoloni et al., 2022), their widespread applications is limited. With recent advancements in stem cell technology, the emergence of a three‐dimensional (3D) bronchoalveolar organoid (BAO) model has provided an alternative in vitro model for investigating lung disease (Chia et al., 2022; Clevers, 2016; Hu et al., 2021; Schutgens & Clevers, 2020; Tran et al., 2022).

Organoids can be categorized based on their fabrication methods into either “engineering‐based organoids”, which encompass organoids derived from embryonic stem cells (ESCs) and induced pluripotent stem cells (iPSCs) (Huang et al., 2014; Jacob et al., 2017; McCauley et al., 2017; Miller et al., 2019), or “non‐engineering‐based organoids”, which including organoids from tissue‐derived primary cells or adult stem cells (ADCs) (Basil et al., 2022; Kadur Lakshminarasimha Murthy et al., 2022; Lim et al., 2023; Nikolić et al., 2017). One of the persistent challenges with iPSC‐derived lung organoid (LO) model, when compared to “non‐engineering‐based organoids”, is the issue of contamination with mesoderm‐lineage or non‐lung endoderm cell types during model generation. Conversely, LO models generated using lung tissue‐derived primary cells maintain a relatively homogenous cell population of the required origin and have served as foundational model for investigating a range of lung diseases (Tindle et al., 2021).

The BAO model primarily consists of cell subtypes of proximal airway, such as basal, ciliated and club cells, while alveolar Type 1 and 2 (AT1 and AT2) cells are limited present. Recently, Zambrano et al. introduced a distal airway differentiation method that induced the formation of nascent alveolar structures after adding factors including IBMX, 8‐bromo‐cAMP, and dexamethasone (Magro‐Lopez et al., 2018). This differentiation strategy has been utilized in various studies to create alveolar organoid (AO) model for investigating change of alveolar epithelia during diverse respiratory diseases (Chiu et al., 2022; Sempere et al., 2022). While morphological and cellular assessments have been employed to validate the differentiated AO model (Chiu et al., 2022; van der Vaart & Clevers, 2021), the limited morphological characters and cellular markers fail to offer a comprehensive landscape of molecular alterations that could elucidate the underlying mechanisms of the biological process or disease progression.

In this study, we reconstructed the BAO and AO model using a differentiation approach previously described (Magro‐Lopez et al., 2018), and confirmed the changes in morphology and cellular composition through the utilization of a previously established AI organoid tracing and image analysis system (Bian et al., 2021) and immune‐fluorescent staining. In addition, the molecular networking alterations, aiming to uncover the mechanisms underlying the observed morphological and functional changes, through proteomic analysis.

## 2 MATERIALS AND METHODS

### 2.1 Establishment of murine BAO model

Male C57BL/6 mice aged 4–6 weeks were procured from Jennio Biotech Company (GuangZhou, China). All animal experiments conducted adhered to the guidelines set forth by the Ethics Committee of Laboratory Animal Center of Nanfang Hospital Southern Medical University and were in in compliance with relevant guidelines of the Chinese government and regulations for the care and use of experimental animals. The approval for these experiments is documented under the encode NFYY‐2020‐0429.

The mice were humanely sacrificed through spinal cord dislocation, a method endorsed by the American Veterinary Medical Association (AVMA) Guidelines for the Euthanasia of Animals. Lung tissues from C57BL/6 mice were used exclusively for the generation of distal airway organoid/BAOs. Upon receipt, the tissues were minced and subjected to enzymatic digestion within a solution comprising dispase II (Roche; #04942078001), DNase I (MilliporeSigma, #10104159001) and collagenase III (Gibco, #17101–015) at 37°C for 2 h. Post‐digestion, the supernatant was filtered with 40 μm cell strainer (JET BIOFIL, #CSS013040) and centrifugated at 200 g for 5 min. The pellet was resuspended in culture media and mixed with Matrigel (Corning, #356231) at a ratio of 1:1.5 (v:v), subsequently, transferred into a 24 well plate at dropwise. Finally, these droplets were allowed to solidify in a 37°C 5% CO_2_ incubator for 10 min and culture medium was added and refreshed every 2–3 days.

In this study, three different culture media, LO‐1 (LO culture medium‐1), LO‐2 (LO culture medium‐2) and EM (Expansion medium) were used and the components of each medium are listed in Table S1.

### 2.2 Conditioned organoid culture model

BAO colonies were dissociated into small cell clusters and resuspended in EM. Following cell counting, the appropriate number of viable cells mixed with Matrigel (Corning, #356231) at a ratio of 1:1.5 (v:v) and subsequently reseeded in the 24‐well plate. The differentiation medium (DM), as described in a previous study (Magro‐Lopez et al., 2018), containing IBMX (Invitrogen, # PHZ1124), 8‐bromo‐cAMP (abcam, #76939–46‐3) and dexamethasone (Solarbio, #ID0170) (Table S1), was added and incubated in 37°C, 5% CO_2_ incubator for 14 days. On the fifth day post‐DM addition, the DM was refreshed by replace half the medium with new fresh medium.

### 2.3 Histology and immunofluorescence

For histology analysis, organoids were fixed with 4% paraformaldehyde (Boster, #AR1068) and embedded in paraffin. Subsequently, 5 μm thick sections were deparaffinized in xylene, hydrated in a graded ethanol series and stained using hematoxylin–eosin, Alcian blue, PAS staining kit (Abcam, #ab245876) following the manufacturer's protocol.

In preparation for immunofluorescence analysis, paraffin sections of the organoids were pre‐blocked with 5% goat serum (solarbio, #SL038) for 10 min at room temperature, following the incubation with primary antibody at 4°C overnight. The following day, stained sections were treated with fluorescent secondary antibodies for 2 h at room temperature. Imaging was conducted using an OLYMPUS IX73 microscope. Details of the antibodies are provided in Table S2.

### 2.4 Total RNA isolation and quantitative RT‐PCR

Total RNA was extracted from organoids using Cell Total RNA Extraction Kit (TRIzol™ Reagent, Life technology) according to the manufacturer's protocol. 1 μg of isolated RNA was applied to synthesize cDNA using FastKing gDNA Dispelling RT SuperMix kit (TIANGEN BIOTECH, #4992226) according to the manufacture's protocol. Quantitative RT‐PCR was performed with SYRB GREEN Realtime PCR Master Mix (TIANGEN BIOTECH, #4992929), with custom primers obtained from GENEWIZ with the primer purity of 80%. The sequence of PCR primers is detailed in Table S3. PCR experiments were performed in triplicates. The expression of target samples was normalized to the internal control β‐actin, and the relative mRNA expression values are calculated as shown in Table S4.

### 2.5 AI based image analysis

To accurately quantify the number of organoids displaying different characteristics, we have implemented an image analysis method based on artificial intelligence. Initially, we identified all organoids from microscopic images by Faster‐Rcnn (Ren et al., 2017), which is a commonly used object detection framework. Subsequently, the identified organoids were inputted into VGG (Simonyan & Zisserman, 2015), a convolutional neural network (CNN) based deep learning framework, for classification into predefined three categories based on their appearance. The proportion of organoids belonging to each category in the image was then calculated.

Practically, to enable organoid detection, we curated an organoid detection dataset. This involved manual annotation of several high‐throughput organoid microscopic images, followed by cropping the original image into multiple image patches of equal size. The Faster‐Rcnn model was trained with the dataset to annotate organoid detection. For organoid classification, we assembled a dataset comprising 100 predefined organoid image patches. The VGG model was trained using the constructed dataset to achieve automatic organoid classification. To improve the predictive accuracy of the models, we initialized the parameters of the aforementioned artificial intelligence models with pre‐training parameters of ImageNet (Bian et al., 2021), and fine‐turned these models with self‐defined datasets.

### 2.6 Sample preparation for proteomic analysis of BAOs

To identify the proteins of BAOs treated with DM and EM, BAOs were harvested at 10 days after DM or EM treatment and analyzed through proteomics analysis. Sample lysis and protein extraction were conducted using SDT buffer (4% SDS, 100 mM Tris–HCl, 1 mM DTT, pH 7.6). Protein quantification was performed using the BCA Protein Assay Kit (Bio‐Rad, USA). The process of protein digestion by trypsin was carried out using the filter‐aided sample preparation (FASP) method. Peptides were desalted using C18 Cartridges and reconstituted in 0.1% formic acid. SDT buffer (4% SDS, 100 mM DTT, 150 mM Tris–HCl pH 8.0) was used with 200 μg of proteins per sample. UA buffer (8 M Urea, 150 mM Tris–HCl pH 8.0) was used to remove detergent, DTT, and low‐molecular‐weight components through repeated ultrafiltration (Microcon units, 10 kD). Iodoacetamide was added to block cysteine residues and incubated for 30 min. Filters were washed with 100 μL UA buffer (3x) and 100 μL 25 mM NH4HCO3 buffer (2x). Trypsin digestion of protein suspensions was performed overnight at 37°C. The peptides in each sample underwent desalting, concentration, and reconstitution in 40 μL of 0.1% formic acid. Peptide content was estimated using UV light at 280 nm.

### 2.7 TMT labeling and strong cation exchange (SCX) fractionation

According to the manufacturer's instructions (Thermo Scientific), the peptide mixture of each sample, measuring 100 μg, was labeled using TMT reagent. Fractionation of labeled peptides was performed using the AKTA Purifier system (GE Healthcare) and SCX chromatography. The dehydrated peptide mixture was rehydrated and acidified using buffer A (10 mM KH_2_PO_4_ in 25% ACN, pH 3.0), then applied to a PolySULFOETHYL 4.6 × 100 mm column (5 μm, 200 Å, PolyLC Inc). A gradient elution was performed at a flow rate of 1 mL/min, starting from 0% and reaching 100% buffer B (containing 500 mM KCl, 10 mM KH_2_PO_4_ in 25% ACN, pH 3.0), with buffer B being subsequently reset to 0% after 60 min. Absorbance at 214 nm was utilized to monitor the elution, with fractions being collected at regular 1‐min intervals. The collected fractions were subjected to desalting using C18 Cartridges (Empore SPE Cartridges C18, standard density) with a bed inner diameter of 7 mm and a volume of 3 mL (Sigma), followed by concentration through vacuum centrifugation.

### 2.8 LC–MS/MS analysis

LC–MS/MS analysis lasting 60/90 min was conducted using a Q Exactive mass spectrometer (Thermo Scientific) that was connected to Easy nLC (Thermo Scientific). The peptides were loaded onto a reverse phase trap column (Acclaim PepMap100, 100 μm × 2 cm, nanoViper C18, Thermo Scientific) connected to the C18‐reversed phase analytical column (Easy Column, 10 cm long, 75 μm inner diameter, 3 μm resin, Thermo Scientific) in 0.1% Formic acid (Buffer A), and separated with a linear gradient of 84% acetonitrile and 0.1% Formic acid (Buffer B) at a flow rate of 300 nL/min controlled by IntelliFlow technology. Positive ion mode was employed during the operation of the mass spectrometer. Acquisition of MS data was performed utilizing a data‐dependent top10 method, whereby the most abundant precursor ions from the survey scan (300–1800 m/z) were dynamically chosen for HCD fragmentation. The target for the Automatic Gain Control (AGC) was set to 3e6, with a maximum inject time of 10 ms. The duration of dynamic exclusion was 40.0 seconds. The survey scans were obtained at a resolution of 70,000 at m/z 200. Additionally, the resolution for HCD spectra was configured to 17,500 at m/z 200, with an isolation width of 2 m/z. The normalized collision energy was set at 30 eV, while the underfill ratio, which indicates the minimum percentage of the target value expected to be achieved at maximum fill time, was defined as 0.1%. The instrument was operated with peptide recognition mode activated. The MASCOT engine (Matrix Science, London, UK) was utilized to search the MS raw data for each sample.

### 2.9 Bioinformatic analysis

Cluster 3.0 (http://bonsai.hgc.jp/~mdehoon/software/cluster/ software.htm) and Java Tree view software (http://jtreeview.sourceforge.net) were used to performing hierarchical clustering analysis. In the process of hierarchical clustering, the Euclidean distance algorithm was employed as the similarity measure, along with the average linkage clustering algorithm that utilizes the centroids of the observations. A heat map was commonly used with the dendrogram as a visual aid.

To predict protein subcellular localization, the researchers utilized CELLO, a multi‐class SVM classification system, which can be accessed at http://cello.life.nctu.edu.tw/. Protein sequences were searched for homologs using NCBI BLAST+ and InterProScan, then annotated with Blast2GO. The GO annotation results were plotted by R scripts.

The annotation process involved blasting the studied proteins against the online Kyoto Encyclopedia of Genes and Genomes (KEGG) database (http://geneontology.org/). This was done to obtain their KEGG orthology identifications, which were subsequently mapped to pathways in KEGG.

Fisher's exact test was used for enrichment analysis with all quantified proteins as background. The Benjamini‐Hochberg correction for multiple testing was additionally used to adjust the obtained *p*‐values. Significance was attributed solely to functional categories and pathways with *p‐*values below the threshold of 0.05.

### 2.10 Western blotting

The standard Western blotting protocol was used. To capture the 45 g of total protein derived from organoids, a PVDF membrane was employed and transferred onto a 15% SDS‐PAGE gel. The membranes were blocked for 60 min using a mixture of 5% skimmed milk and TBS‐T. Following an overnight incubation at 4°C with primary antibodies against AQP5 (diluted 1:1000, Abcam, UK, #ab305303), SFTPA (diluted 1:1000, Abcam, UK, #ab180865), P63 (diluted 1:1000, Abcam, UK,#ab124762), FOXK1 (diluted 1:1000, Abcam, UK, #ab309510), SMARCC1 (diluted 1:1000, Abcam, UK, #ab305037), the membranes were exposed to a secondary antibody, goat anti‐rabbit IgG‐HRP (diluted 1:5000, Jackson lmmunoResearch, USA, # 111–035‐003), for 120 min at room temperature. The membranes were then shaken and rinsed three times for 15 min with TBS‐T before being imaged through Chemiluminescent Substrate System (Thermo Scientific, USA).

## 3 RESULTS

### 3.1 Morphology characteristics of murine BAOs

**FIGURE 1:**
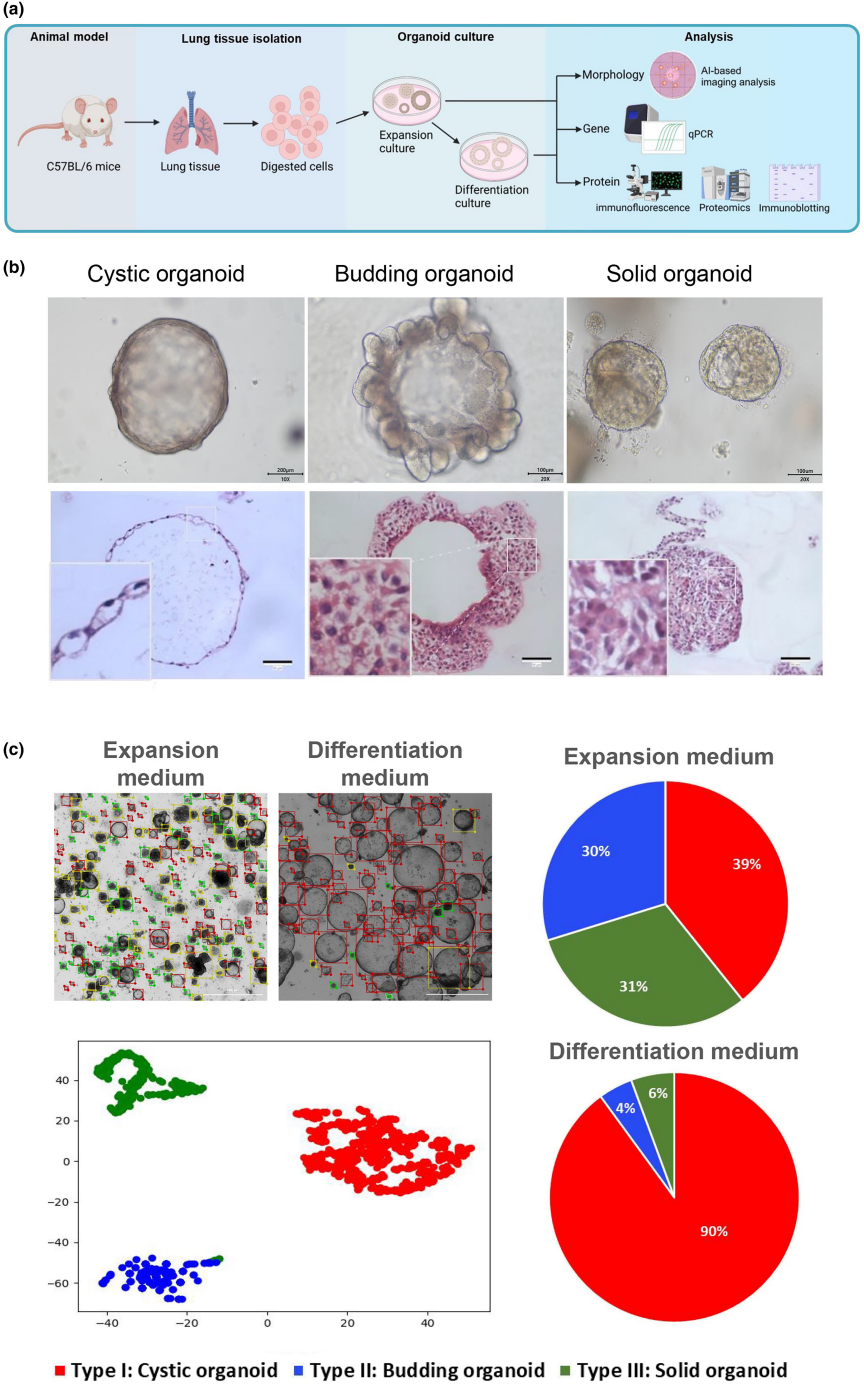
The morphology changes of murine lung organoid after differentiation. (a) Schematic of generation and analysis of the (un)differentiated murine lung organoids; (b) representative phenotypes of undifferentiated murine lung organoids; *n* = 3 per group. (c) The composition analysis of the undifferentiated and differentiated murine lung organoid based on AI morphology characterization; *n* = 3 per group.

To generate a fast, robust and sustainable BAO culture system, we refined previously reported LO culture media (Magro‐Lopez et al., 2018), which we termed as EM. The EM facilitated a consistent and accelerated growth environment for murine BAOs, allowing for extended culture up to 20 passages (Figure S1). The validation workflow post differentiation of BAOs is shown in Figure 1a.

Using our previously developed AI organoid tracing, capture and analysis technique (Bian et al., 2021), we examined the morphological attributes of BAOs (Table S5). Based on their morphological features, the BAOs were categorized into three distinct types: Type I cystic organoids, Type II budding organoids and Type III solid organoids (Figure 1b). To determine the morphological change post‐differentiation, the organoid composition before and after addition of DM was analyzed. Prior to the addition of the DM, the BAOs exhibited a nearly equal distribution among three organoid type. However, post‐treatment with the DM, the organoids predominantly displayed a Type I cystic structure (Figure 1c).

### 3.2 Molecular and genetic profile of murine BAOs

**FIGURE 2:**
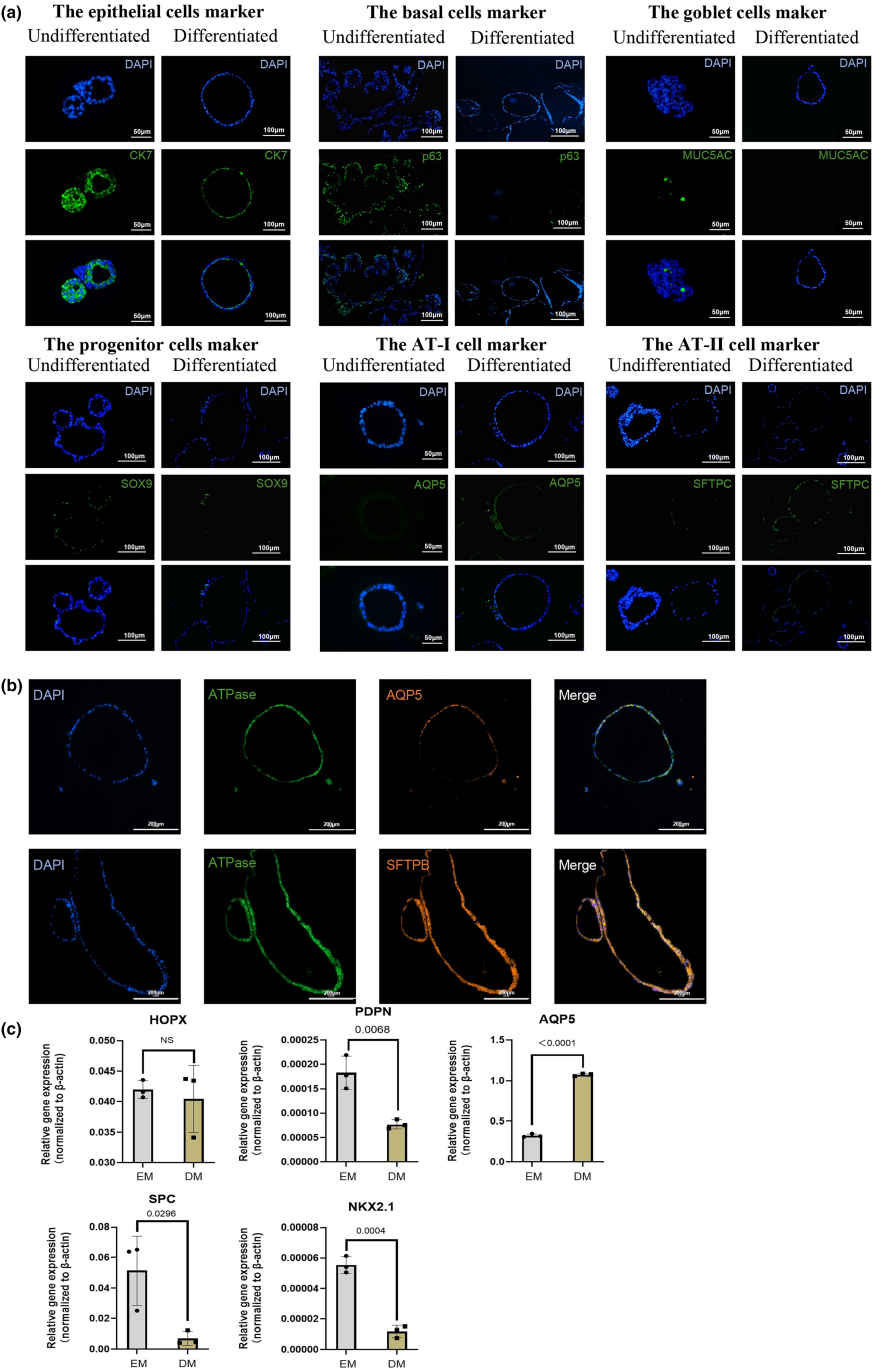
The molecular characterization of the (un)differentiated murine lung organoids. (a) IF images of the expression of epithelial cells marker (cytokeratin 7, CK7), basal cells marker (P63), goblet cells maker (MUC5AC), progenitor cells maker (SOX9), AT‐I cell marker (AQP5), AT‐II cell marker (SFTPC) on (un)differentiated cells; (b) the co‐localization of the ATPase, AQP5 and SFTPB on bronchoalveolar organoid after differentiation; (c) the alteration of mRNA expression of lung progenitor cell marker(NKX2.1), AT‐I cell markers (Hopx, PDPN, AQP5) and AT‐II cell marker (SPC) after differentiation. *n* = 3 per group; Unpaired *t*‐test is used for the statistical comparison between the two groups in (a–e); NS: no significant differences were recorded.

To further elucidate cellular composition of BAOs both pre‐ and post‐differentiation, the expression levels of various cell markers were investigated. These included the progenitor cells maker Sex‐determining region Y (SRY)‐box 9 protein (SOX9), the epithelial cell marker cytokeratin 7 (CK7), the basal cell markers P63, the goblet cells maker Mucin 5 AC (MUC5AC), the Type I alveolar cell (AT‐I) marker aquaporin 5 (AQP5) and the Type II alveolar cell (AT‐II) marker Surfactant Protein C (SFTPC) were examined (Christin et al., 2020; Rockich et al., 2013; Scott et al., 2010; Vaughan et al., 2015; Zuo et al., 2015) (Figure 2a).

The overall positive staining results for CK7 and SOX9 indicated the presence of a subset of rapidly proliferating epithelial cells possessing stem cell properties under both culture conditions. The notable expression of p63 and Muc5AC in EM condition, but not in DM condition, suggested that goblet cells and basal cells were predominantly present during expansion but not post‐differentiation induction. In addition, the expression of the AT1 marker AQP5 were observed in organoid cultured under both EM and DM conditions, and the AT2 marker SFTPC was mainly expressed on organoid cultured in both of EM and DM condition.

To further analyze the characteristics of the alveolar cells post‐differentiation, the expression of AT cell markers in conjunction with the ATPase, a key regulator for the fluid clearance of alveolar cells, were accessed (Ridge et al., 2003). Co‐localization of ATPase with both AQP5 and SFTPB was observed (Figure 2b). This finding suggested that both AT1 and AT2 cells likely possess the capability to perform fluid clearance functions.

In summary, these staining data suggest that the generation and expansion of organoid led to an enrichment of epithelial cells with proliferation capability, while the DM culture condition appeared to promote the forming of AT cells, but not other types of lung cell lineages.

To investigate the genetic alteration of alveolar cells following differentiation, we examined the mRNA expression of early lung progenitor cells and diverse alveolar cell markers. Consistent with the results of immune staining, a notable reduction in the expression of the early lung progenitor marker *nkx2.1* was observed following differentiation, along with a decrease in the expression of the early stage AT1 cell marker *pdpn* and the AT2 cell marker *sftpc* (Figure 2c). In contrast, increasing genetic expression level of mature AT1 cell markers *aqp5* was increased under DM condition (Figure 2c), suggested the maturation of AT1 cells. These observations may suggest a distinct regulation mechanism or activation time point between the RNA and protein expression.

### 3.3 Proteomic profiling of murine BAOs

**FIGURE 3:**
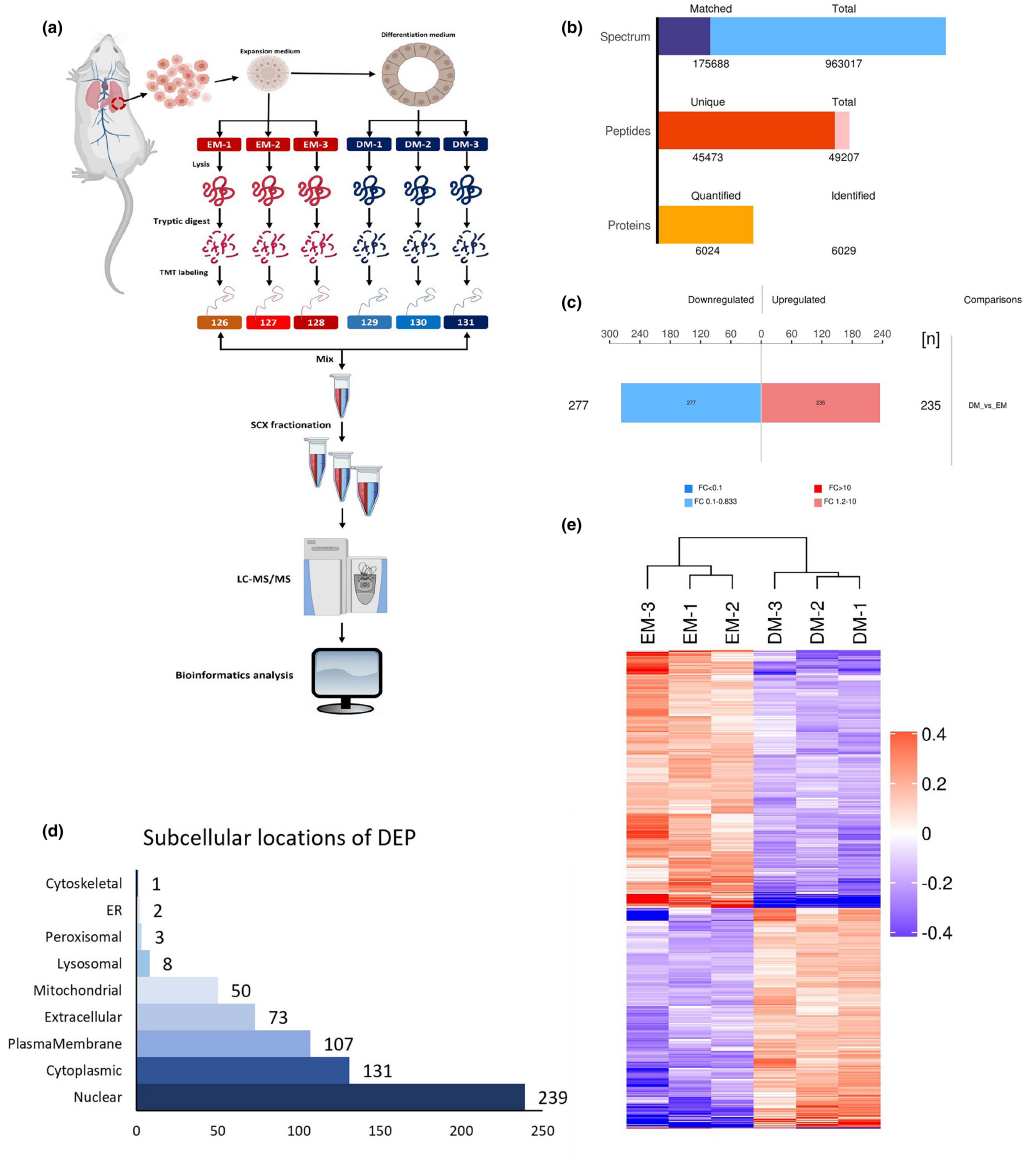
The alteration of proteomic profile of murine lung organoid after differentiation is not identical to the development process. (a) Schematic of the proteomic analysis process. *n* = 3 per group. (b) The characteristics of detected proteins derived from murine lung organoids. (c) The alteration of the proteomic profile of murine lung organoid after differentiation. (d) The characteristics of altered proteomic profile. (e) Heatmap of DEPs between EM and DM groups.

The proteomic alterations of pre‐ and post‐differentiation, were assessed through a liquid chromatography–tandem mass spectrometry (LC–MS/MS) analysis that incorporated tandem mass tag (TMT) labeling. The proteomic profiles of organoids from both EM and DM culture conditions were analyzed (Figure 3a) (Table S6). In total, a sum of 175,688 matched spectra were identified, resulting in the detection of 45,473 unique peptides. A total of 6029 proteins can be generated from these peptides, with 6024 of them being quantified (Figure 3b). According to the comparative analysis, there were 512 differently expressed proteins (DEPs) in organoids after the induction of alveolar‐like morphology, compared to organoids before the morphology change. These DEPs exhibited a fold change >1.2 and a *p* < 0.05. Among them, 235 proteins were up‐regulated and 277 proteins were down‐regulated (Figure 3c, e).

The DEPs were classified according to their cellular localization, with nuclear proteins having the highest representation, followed by cytoplasmic and plasma membrane proteins. The extracellular compartment (Figure 3d) exhibited the lowest protein count. The observation implied that the molecular alterations during the DM culture primarily involved intracellular proteins.

### 3.4 In silico functional analysis of altered proteomic profile of murine BAOs after differentiation

**FIGURE 4:**
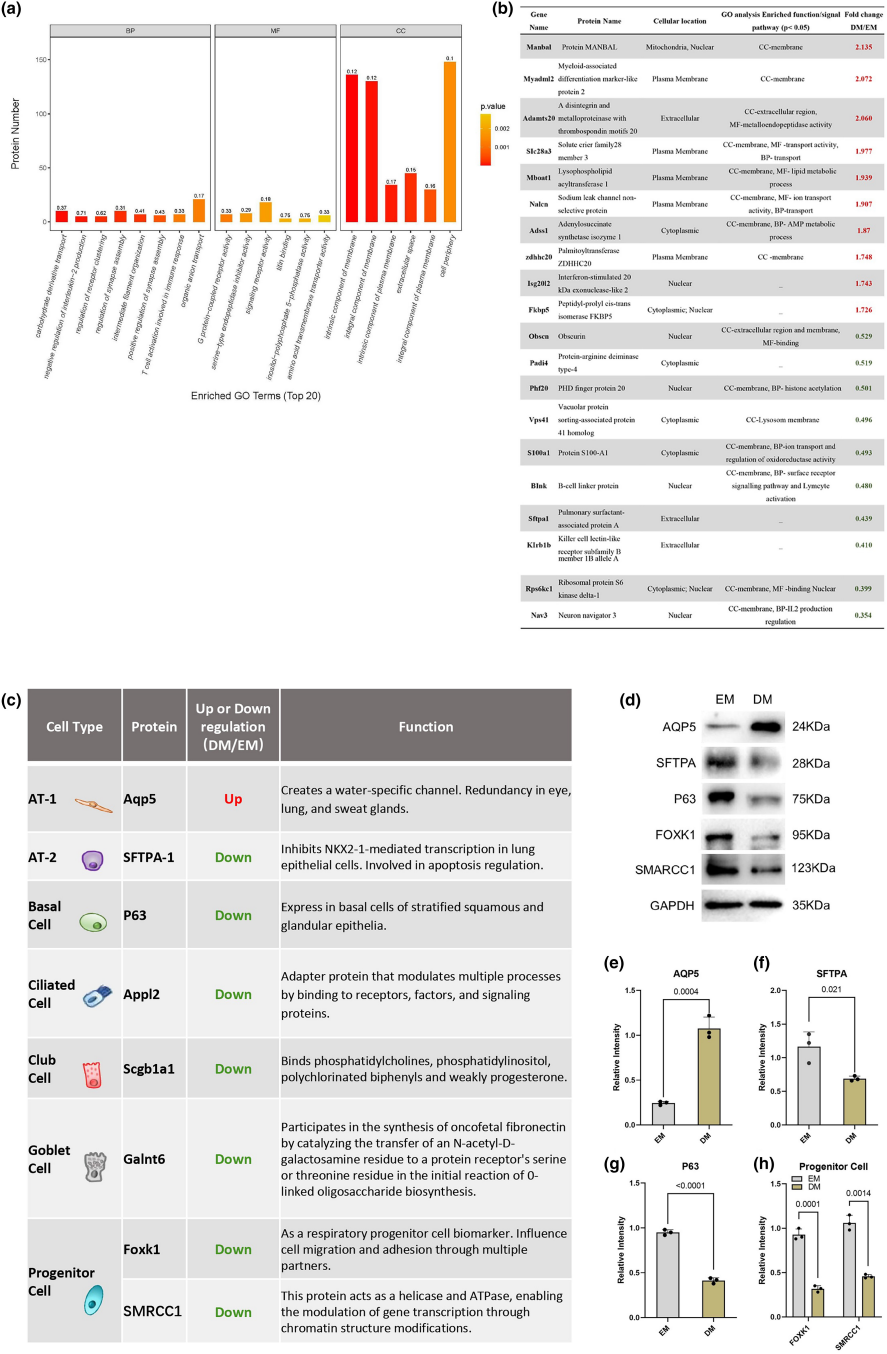
The functional analysis of the proteomic alteration of murine lung organoid after differentiation. (a) A summary of different expression proteins (DEPs) associated with biological process, molecular function and cellular component in gene ontology (GO) analysis. (b) The top 10 significantly up‐regulated and down‐regulated DEPs and it related function or signal pathways. (c) A list of proteins that have the potential to form a molecular signature identifying alveolar Organoid. (d) Western blot of cell‐specific different expression proteins. Quantified analysis of the expression of critical proteins included AQP5 expression (e), SFTPA (f), P63(g), FOXK1 and SMRCC1(h), the *p*‐value of Student's *t*‐test were displayed on the bar charts.

To gain a glimpse into the overall functional alterations in the protein profile, bioinformatic analyses were carried out, specifically gene ontology (GO) annotation, using the proteomic profiling data from both BAO and AO. The GO enrichment analysis provides an indication of the biological process (BP), molecular function (MF) and cellular component (CC) in which the DEPs were enriched, and the number of significant enriched BP GO terms, MF GO terms and CC GO terms were 279, 88 and 36, respectively. Abounded number of DEPs enriched in BP was related protein transport, while main part of DEPs enriched in MF analysis was associated with signaling transduction. In line with proteomic localization analysis outcome, CC enrichment analysis data demonstrated that most DEPs were either part of intracellular components like organelles or (plasma) membrane, but not extracellular components (Figure 4a).

In order to analyze the biological features of the most diverse DEPs, we specifically focused on the top 10 significantly up‐ and down‐regulated DEPs (Figure 4b, Table 1). Among the up‐regulated DEPs, the number of DEPs enriched in G.O. terms CC, MF and BP were eight, four and three, respectively. Only two DEPs, Interferon‐stimulated 20 kDa exonuclease‐like 2 (ISG20L2) and Peptidyl‐prolyl cis‐trans isomerase FKBP5 were not found in significantly enriched G.O. terms (Table S7). The enrichment analysis data demonstrated that all DEPs were enriched in plasma membrane formation of CC GO analysis term, while the main biological pathways in which they were enriched are signal/molecule transport and metabolic processes (Figure 4b, Table 1). An example was the sodium leak channel non‐selective protein (Nalcn), which locates on the cell plasma membrane and is important to maintain the normal respiratory rhythm and contribute to alveolar fluid clearance under physiological conditions (Lu et al., 2007; Zhang et al., 2017). On the contrary, among the down‐regulated DEPs, the number of DEPs enriched in G.O. terms CC, MF and BP were seven, two and four, respectively. Three DEPs, Protein‐arginine deiminase Type‐4 (PADI4), Pulmonary surfactant‐associated protein A (SFTPA1) and Killer cell lectin‐like receptor subfamily B member 1B allele A (KLRB1B) were not found in significantly enriched G.O. terms (Table S7). These down‐regulated DEPs were enriched in nuclear or organelle membrane formation process, while the main biological pathways in which they were enriched are histone acetylation and lymphocyte activation signaling pathways (Figure 4b, Table 1). Taken together, although these data did not indicate an airway tissue‐specific process but did indicate enhanced general biological processes including membrane formation and metabolic process, and reduced processes such as organelle membrane formation and multi cell type signaling activity.

**TABLE 1:** The top ten significantly upregulated and downregulated DEPs and it related function or signal pathways.

Gene name	Protein name	Cellular location	GO analysis enriched function/signal pathway (*p* < 0.05)	Fold change DM/EM	*T*‐test *p* value
Manbal	Protein MANBAL	Mitochondria, nuclear	CC‐membrane	2.135	0.01223
Myadml2	Myeloid‐associated differentiation marker‐like protein 2	Plasma membrane	CC‐membrane	2.072	0.00916
Adamts20	A disintegrin and metalloproteinase with thrombospondin motifs 20	Extracellular	CC‐extracellular region, MF‐metalloendopeptidase activity	2.060	0.00830
SIc28a3	Solute crier family28 member 3	Plasma membrane	CC‐membrane, MF ‐transport activity, BP‐ transport	1.977	0.01532
Mboat1	Lysophospholipid acyltransferase 1	Plasma membrane	CC‐membrane, MF‐ lipid metabolic process	1.939	0.00018
Nalcn	Sodium leak channel non‐ selective protein	Plasma membrane	CC‐membrane, MF‐ ion transport activity, BP‐transport	1.907	0.00150
Adss1	Adenylosuccinate synthetase isozyme 1	Cytoplasmic	CC‐membrane, BP‐ AMP metabolic process	1.87	0.00124
zdhhc20	Palmitoyltransferase ZDHHC20	Plasma membrane	CC ‐membrane	1.748	0.04003
Isg20l2	Interferon‐stimulated 20 kDa exonuclease‐like 2	Nuclear	_	1.743	0.00272
Fkbp5	Peptidyl‐prolyl cis‐trans isomerase FKBP5	Cytoplasmic; nuclear	_	1.726	0.00001
Obscn	Obscurin	Nuclear	CC‐extracellular region and membrane, MF‐binding	0.529	0.00057
Padi4	Protein‐arginine deiminase type‐4	Cytoplasmic	_	0.519	0.02049
Phf20	PHD finger protein 20	Nuclear	CC‐membrane, BP‐ histone acetylation	0.501	0.00545
Vps41	Vacuolar protein sorting‐associated protein 41 homolog	Cytoplasmic	CC‐Lysosom membrane	0.496	0.01783
S100a1	Protein S100‐A1	Cytoplasmic	CC‐membrane, BP‐ion transport and regulation of oxidoreductase activity	0.493	0.00218
BInk	B‐cell linker protein	Nuclear	CC‐membrane, BP‐ surface receptor signalling pathway and Lymcyte activation	0.480	0.00178
Sftpa1	Pulmonary surfactant‐ associated protein A	Extracellular	_	0.439	0.00746
KIrb1b	Killer cell lectin‐like receptor subfamily B member 1B allele A	Extracellular	_	0.410	0.00019
Rps6kc1	Ribosomal protein S6 kinase delta‐1	Cytoplasmic; nuclear	CC‐membrane, MF ‐binding Nuclear	0.399	0.01061
Nav3	Neuron navigator 3	Nuclear	CC‐membrane, BP‐IL2 production regulation	0.354	0.02211

Next, whether the altered protein expression could explain the morphological alterations during DM treatment were studied. Since cell junction proteins is crucial for the architecture of the tissue, we analyzed the DEPs of cell junction and related molecular and functional changes (Table S8) (Uhlén et al., 2005). Generally, the cell junction proteins were altered from bronchus status to alveolar. For example, APBB1IP, SDCBP and PDIM2, which are more expressed in alveolar than bronchus, were increased in DM. In contrast, proteins have lower expression levels in alveolar, such as ABI2, ADGRL3, and OBSCN, were significantly declined during the DM treatment. Among these proteins, a few of them are related to the respiratory functions. These data indicate that altered cell junction protein during DM may explained at least part for the morphological and functional changes of BAO.

The purpose of this investigation was to assess whether the in‐vitro differentiation condition used in this study resulted in comparable molecular changes. In order to accomplish this, we performed a comparative analysis of the fundamental molecular changes that indicate the condition of respiratory epithelial cells, alveolar cells, and progenitor cells (Figure 4c, Table 2). The AQP5, serving as an indicator of AT1 cell, exhibited a notable increase under DM treatment, consistent with a prior study that investigated molecular networks in murine lung development using tissues from embryonic to early adult stages (Moghieb et al., 2018). In contrast, treatment with DM displayed a decrease in the expression of SFTPA, a marker specifically associated with AT‐2 cells. The molecular markers associated with basal cell, ciliated cell, club cell, and goblet cell showed a decrease in expression following the process of differentiation. In terms of the progenitor cells, the FOXK1 and SMRCC1 were both decreased, indicating the maturation of BAOs. The alternative trends of key DEPs have been confirmed through Western blotting, with the observed tendencies aligning with the results obtained from LC–MS/MS based proteomics analysis (Figure 4d–h) (Figure S2).

**TABLE 2:** A list of proteins that have the potential to form a molecular signature identifying alveolar organoid.

	Protein name	Up‐ or down‐regulation in DM	Function
AT‐1 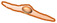	**Aqp5**	**Up**	Creates a water‐specific channel. Redundancy in eye, lung, and sweat glands.
AT‐2 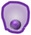	**SFTPA‐1**	**Down**	Inhibits NKX2‐1‐mediated transcription in lung epithelial cells. Involved in apoptosis regulation.
Basal cell 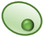	**P63**	**Down**	Express in basal cells of stratified squamous and glandular epithelia.
Ciliated cell 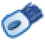	**Appl2**	**Down**	Adapter protein that modulates multiple processes by binding to receptors, factors, and signaling proteins.
Club cell 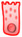	**Scgb1a1**	**Down**	Binds phosphatidylcholines, phosphatidylinositol, polychlorinated biphenyls and weakly progesterone.
Goblet cell 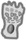	**Galnt6**	**Down**	Participates in the synthesis of oncofetal fibronectin by catalyzing the transfer of an *N*‐acetyl‐D‐galactosamine residue to a protein receptor's serine or threonine residue in the initial reaction of 0‐linked oligosaccharide biosynthesis.
Progenitor cell 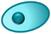	**Foxk1**	**Down**	Mediates effects on cell migration and adhesion through its different partners. As a biomarker for the respiratory progenitor cell.
	**SMRCC1**	**Down**	This protein acts as a helicase and ATPase, enabling the modulation of gene transcription through chromatin structure modifications.

### 3.5 The molecular network alteration of murine BAOs after differentiation

**FIGURE 5:**
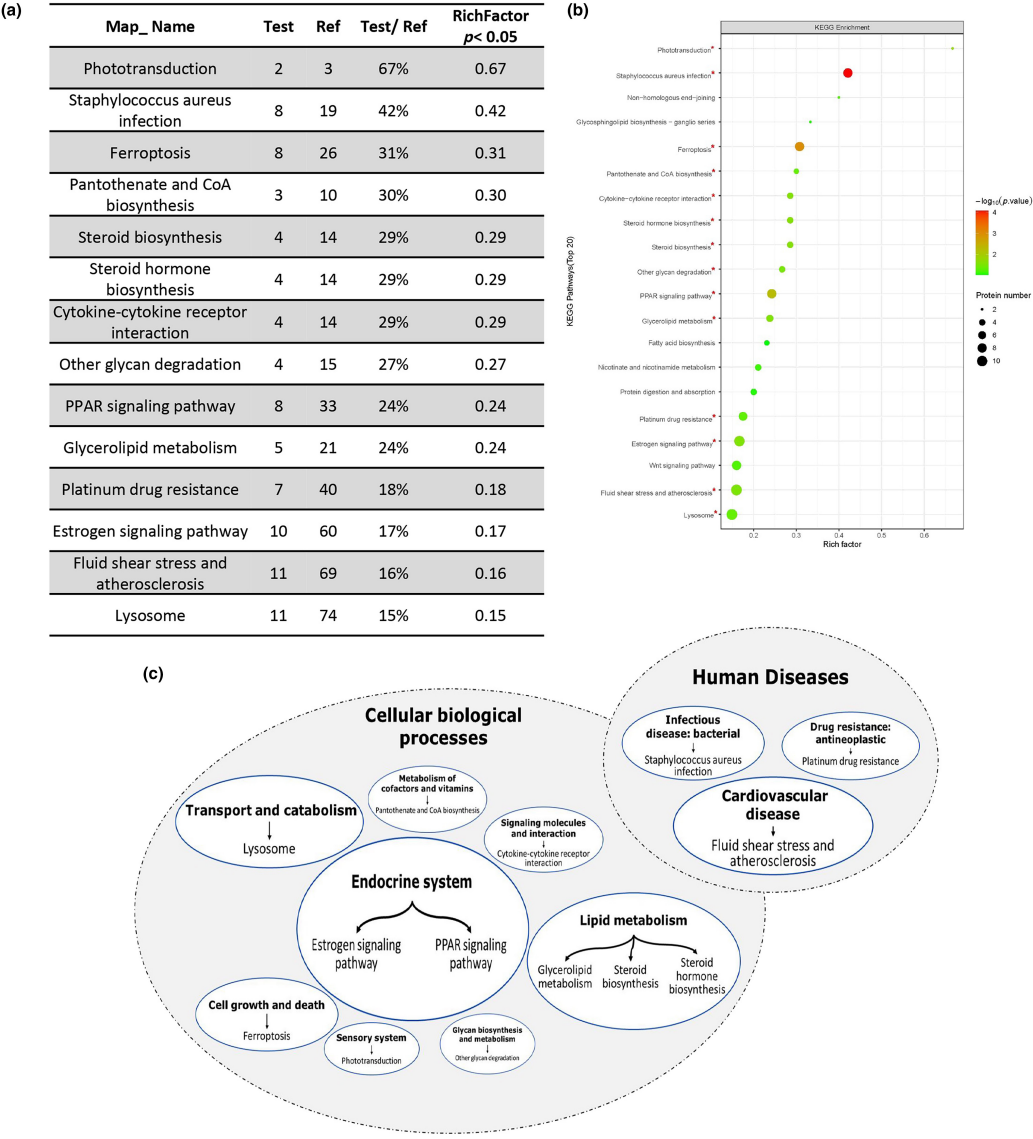
KEGG analysis of DEPs. (a) A list of proteins that have the potential to form a molecular signature identifying alveolar organoid. (b) A scatter plot of KEGG pathway enrichment analysis of DEP; (c) an overview of significantly enriched KEGG pathways and their association.

A KEGG pathway enrichment analysis was carried out on all DEPs to anticipate the modifications in signaling pathways after differentiation induction. The KEGG enrichment terms for all DEPs are displayed in Figure 5a (Table 3), featuring the top 20 terms. Furthermore, Figure 5b provides a comprehensive summary of the 14 significantly enriched pathways and their respective connections. The majority of the enriched pathways were linked to cellular biological processes, with the exception of three pathways related to human diseases: “staphylococcus aureus infection”, “platinum drug resistance”, and “fluid shear stress atherosclerosis”.

**TABLE 3:** An overview of significantly enriched KEGG pathways and their association.

Map_ name	Test	Ref	Test/ref	richFactor *p* < 0.05
Phototransduction	2	3	67%	0.67
Staphylococcus aureus infection	8	19	42%	0.42
Ferroptosis	8	26	31%	0.31
Pantothenate and CoA biosynthesis	3	10	30%	0.30
Steroid biosynthesis	4	14	29%	0.29
Steroid hormone biosynthesis	4	14	29%	0.29
Cytokine‐cytokine receptor interaction	4	14	29%	0.29
Other glycan degradation	4	15	27%	0.27
PPAR signaling pathway	8	33	24%	0.24
Glycerolipid metabolism	5	21	24%	0.24
Platinum drug resistance	7	40	18%	0.18
Estrogen signaling pathway	10	60	17%	0.17
Fluid shear stress and atherosclerosis	11	69	16%	0.16
Lysosome	11	74	15%	0.15

An extensive analysis revealed a notable resemblance between the composition of DEPs in these pathways and those in other pathways (Table S9). As an illustration, seven out of eight differentially expressed proteins (DEPs) that were enriched in the “staphylococcus aureus infection” pathway was also observed in the “Estrogen signaling pathway”. It is important to mention that the majority of these proteins were identified as keratins, which are part of a group of structural proteins. In addition to the high similarity of the DEP pattern, it is possible that other DEPs may play a role in the biological processes associated with the indicated pathway. DEPs such as Top2a, Rev3l, Bad, and Slc31a1, which are grouped in the “platinum drug resistance” pathway, have been identified as being associated with cell survival and active molecular transport.

The pathways related to cellular biological processes that have been enriched can be broadly categorized as metabolic processes, including the “PPAR signaling pathway”, “glycerolipid metabolism”, “steroid biosynthesis”, and “steroid hormone biosynthesis”, as well as cell fate determinative pathways such as the “estrogen signaling pathway”, “glycerolipid metabolism”, and “ferroptosis”, and transport pathways like lysosome (Figure 5c).

The online KEGG database (http://www.genome.jp/kegg/) was utilized for additional analysis of the potential impact of DEPs on the enriched pathways. Although the limited involvement of selected DEPs in certain enriched biological pathways may not accurately reflect the current cellular condition, the aggregation of DEPs in affected reaction chains within other biological pathways does indicate a potential change outcome (See Table S9).

As an illustration, differentially expressed proteins (DEPs) that are enriched in the “PPAR signaling pathway” have the potential to influence the regulatory mechanisms involved in lipid metabolism (Table S9). Within the group of differentially expressed proteins (DEPs) enriched in the “PPAR signaling pathway”, the expression level of FABPs, which are general transporter proteins responsible for transporting diverse fatty acids and related active lipids, showed enhancement. Moreover, the expression of lipid transporter protein, PLTP, and Me1, which are involved in lipogenesis, exhibited a decrease, while the expression of ACS, a protein associated with fatty acid metabolism, showed an increase. The results indicated that the treatment of DM led to the activation of an intricate network of lipid metabolism regulation pathways, which were partially reflected in the enriched pathways such as “glycerolipid metabolism”, “steroid biosynthesis”, and “steroid hormone biosynthesis”.

Another example was the enriched “ferroptosis pathway”, significantly up‐regulated of components associated with glutathione synthesis including SLC3A2 and SLC7A11, cystine transporter units, and GCLC, the catalytic component of the first rate‐limiting ligase of glutathione synthesis were observed (Franklin et al., 2009) (Table S9). Furthermore, there was an observed increase in Ferritin, which is a crucial protein responsible for regulating iron storage and maintaining iron homeostasis. The results indicated the activation of iron regulation mechanisms that enhance cellular iron storage and suppress the cellular ferroptosis process.

## 4 DISCUSSION

In this study, we revisited the BAO and AO models under established culture conditions, confirming anticipated morphological changes and shifts in cell subpopulations induced by differentiation media. The results have unveiled that molecular changes associated with morphological alterations are accompanied with cellular variations. These findings suggest the existence of a potential molecular signature that could differentiate between differentiated AO and BAO models. The challenges in acquiring human distal airway tissues necessitated the use of murine terminal lung tissue in the development of the BAO model. This approach successfully increased productivity while also reducing the need for animal testing. The morphological features of BAOs were thoroughly examined using our team's AI‐based organoid detection system. By employing this AI framework, the conversion of BAO image data into quantifiable digital metrics was achieved objectively, without causing any disruption to the cultural process. Through the implementation of this approach, the reliance on experimental animals was reduced and a more precise quantification and classification of BAOs was achieved by examining morphological disparities (Bian et al., 2021).

Previous studies have presented conflicting data on the expression of alveolar cell marker proteins in differentiated models, with some reporting no significant changes under DM treatment, while others indicating an increase (Chiu et al., 2022; Magro‐Lopez et al., 2018). In our research, the decrease in nkx2.1 (TTF‐1) mRNA, a marker for early lung progenitor cells, suggests the onset of differentiation (Matsubara et al., 2017; McCauley et al., 2017). Despite varied mRNA expression results for different alveolar cell markers after DM treatment, immunofluorescence staining showed a shift toward alveolar‐like structural changes, aligning with functional studies of AOs (Chiu et al., 2022).

Elevated levels of the AT‐I marker protein and reduced levels of other cell type markers further endorse alveologenesis. The co‐localization of ATPase with AT cells validates the functional integrity of AO organoids, providing additional proof of induced alveologenesis following DM treatment (Ridge et al., 2003). The sustained expression of CK5(+) post‐DM treatment may indicate a basal cell subset with stem‐like characteristics crucial for lung regeneration (Zuo et al., 2015). These results highlight the importance of a comprehensive analytical approach to validate in vitro models beyond genetic characterization.

Investigating molecular heterogeneity can enhance the potential applications of organoid models (Sachs et al., 2019; van der Vaart & Clevers, 2021). The proteomic profiles of BAOs and AOs did not reflect protein expression patterns in distal airway development (Moghieb et al., 2018), suggesting that this model may not accurately represent distal airway biological process of fetal development. To illustrate, the AT‐II cells, characterized by SFTPA, demonstrated a reduction during DM treatment, whereas SFTPA showed an elevation during lung development (Moghieb et al., 2018). Conversely, the rise in AT1 cells (identified by AQP5) may differ from AT2 cells (Aspal & Zemans, 2020), potentially elucidating the decline in SFTPA and rise in AQP5 following DM treatment. The GO annotation demonstrated a variety of modifications in biological processes and molecular functions that were induced by DM treatment, leading to changes in protein expression. The presence of DM resulted in a decrease in the differentiation potential of other cell types, including basal cell (identified by P63) (Li et al., 2023) and progenitor cell (FoxK1 and SMRCC1). This observation indicates that DM has the ability to initiate stem cell differentiation and facilitate functional maturation. Individual DEP can fall under multiple GO terms simultaneously. For example, α‐mannosidase 2 (Man2a1), essential for alveolar formation (Akama, Nakagawa et al. 2006), was categorized under both the “integral component of membrane” and “respiratory gaseous exchange” GO terms. These phenomena suggest that identifying proteins with specific expression patterns could distinguish differentiated AOs.

KEGG pathway analysis offers insights into cellular high‐level functions (Kanehisa et al., 2016). But limited proteins in pathways can diminish analysis outcomes. However, the analysis's significance may be diminished by the scant number of proteins identified within certain pathways. This limitation may identify unrelated pathways, like “phototransduction” and “Staphylococcus aureus infection”. Proteins in the “ferroptosis pathway” are involved in iron transport and storage. Increased expression aids in γ‐glutamyl‐peptide synthesis and prevents glutamate accumulation, protecting against ferroptosis. While Increased expression aids in γ‐glutamyl‐peptide synthesis and prevents glutamate accumulation, protecting against ferroptosis (Dixon et al., 2014). The role of these proteins in the “ferroptosis pathway” requires further investigation.

This study highlights the importance of distinguishing between pre‐ and post‐interruption states through a detailed phenotypical and molecular profile of the BAO model. Proteomic detection methods' technical constraints require cautious data interpretation. An AI image analysis system can link morphological changes and molecular shifts. Robust validation systems are vital for identifying specific biomarkers in the BAO model, presenting a promising molecular signature for AO identification.

## AUTHOR CONTRIBUTIONS

Yan Yu contributed to the experimental design, data analysis and manuscript preparation. Min Huang contributed to the experimental design, Bin Zheng, Zexin Chen and Junlang Li contributed to experiments conduction and data analysis. Gang Li is the corresponding author, and contributed to study design and the manuscript edition. All authors read and approved the final manuscript.

## CONFLICT OF INTEREST STATEMENT

The authors declare no potential conflicts of interest with respect to the research, authorship, and publication of this article.

## 5 ETHICS STATEMENT

The purpose, procedures, and risks associated with the study were explained to all participants and their written informed consent was obtained before conducting the experiment. The experimental protocol was approved by the Laboratory Animal Ethics Committee of Nanfang Hospital (No. NFYY‐2020‐0429).

## Supporting information


Figure S1.

Figure S2.

Table S1.

## Data Availability

Refined data are available in the supplemental tables and figures. The mass spectrometry proteomics data have been deposited to the ProteomeXchange Consortium http://proteomecentral.proteomexchange.org via the iProx partner repository (Boes et al., 2021; Ma et al., 2019) with the dataset identifier PXD039096 Additional data and details may be available upon request from the authors.
