# Supplementary material for: Molecular distinctions of bronchoalveolar and alveolar organoids under differentiation conditions
Source: Physiol Rep. 2024 Jun 2;12(11):e16057. doi: 10.14814/phy2.16057 (PMC11144550; doi:10.14814/phy2.16057)
Supplement: Supplementary file 1 — Figure S1. [file PHY2-12-e16057-s002.pdf]

Supplemental figure 1

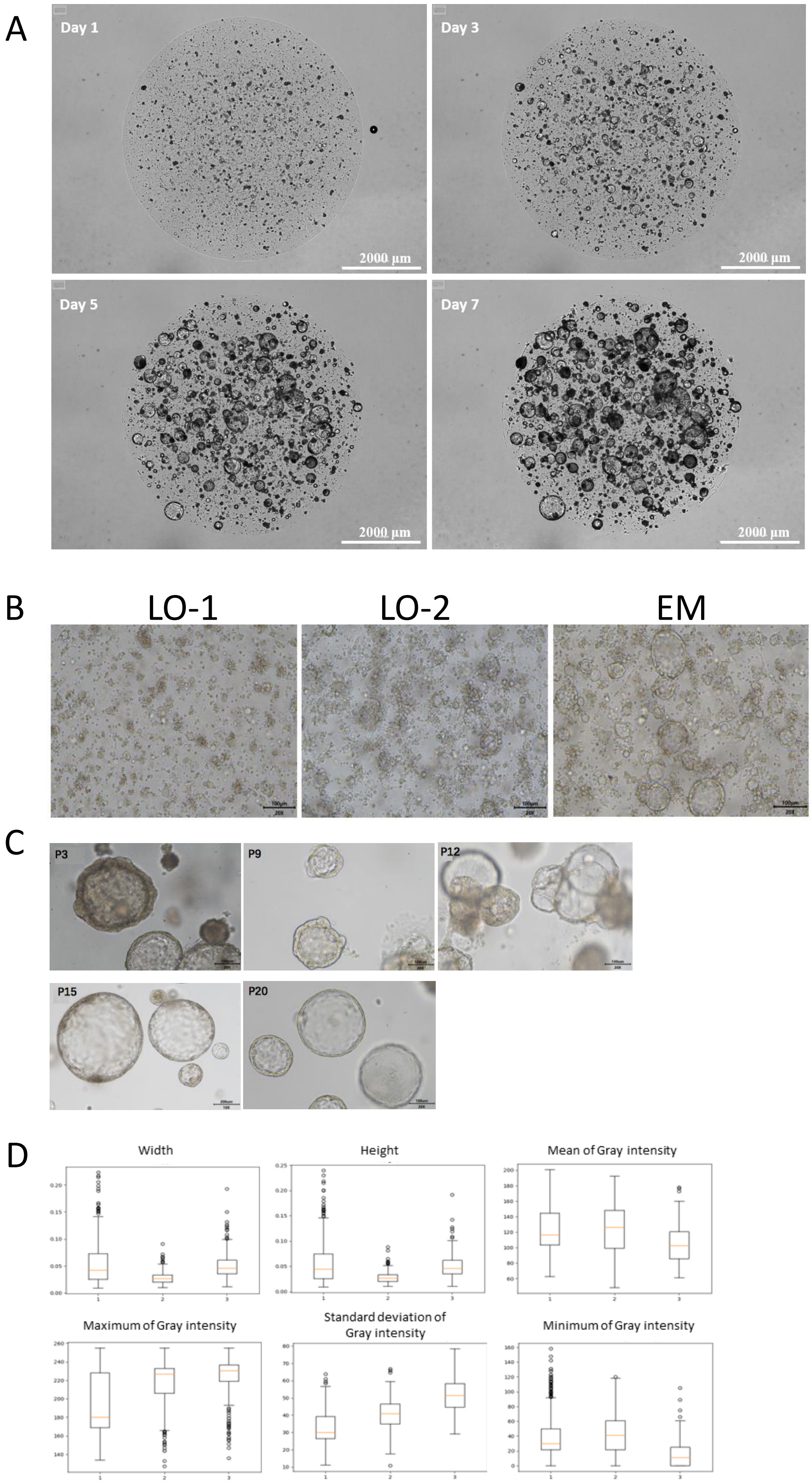

Supplemental figure 1. The optimized expansion culture medium (EM) provided a stable, fast-growing and long-term culture condition for the murine bronchioalveolar organoids. A) The representative images of bronchioalveolar organoids using EM on day 1, 3,5 and 7. B) the representative images of bronchioalveolar organoids using previous reported culture medium LO-1 and LO-2, and EM; C) The representative images of bronchioalveolar organoids on indicated passages. D) The parameters of AI based image analysis.
