# Supplementary material for: Molecular distinctions of bronchoalveolar and alveolar organoids under differentiation conditions
Source: Physiol Rep. 2024 Jun 2;12(11):e16057. doi: 10.14814/phy2.16057 (PMC11144550; doi:10.14814/phy2.16057)
Supplement: Supplementary file 2 — Figure S2. [file PHY2-12-e16057-s003.pdf]

Supplemental figure 2

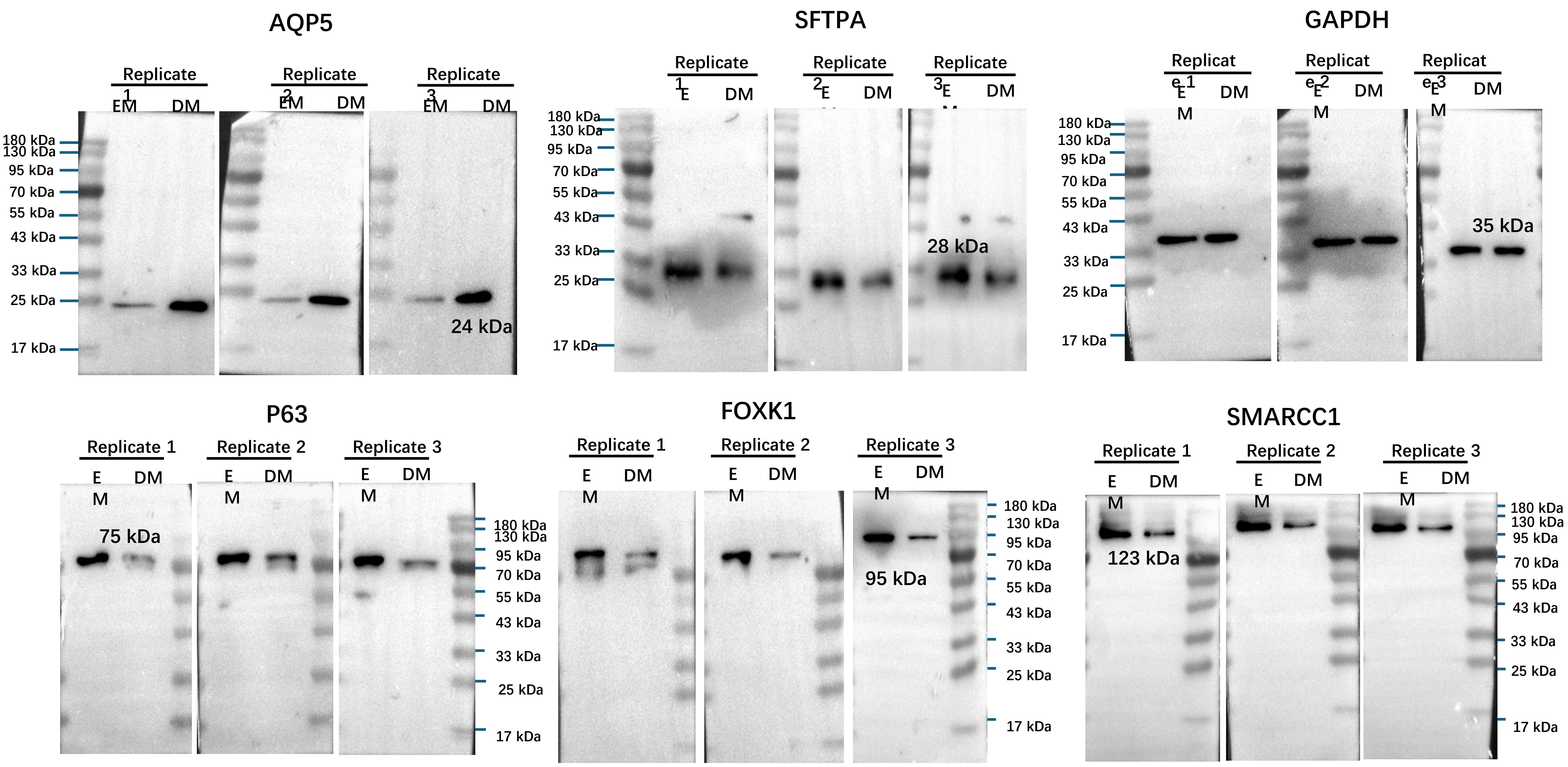

Supplemental figure 2. Western blot of cell-specific different expression proteins. included AQP5 (A), SFTPA (B), GAPDH(C), P63(D), FOXK1 (E) and SMRCC1(F).
