# Supplementary material for: Molecular distinctions of bronchoalveolar and alveolar organoids under differentiation conditions
Source: Physiol Rep. 2024 Jun 2;12(11):e16057. doi: 10.14814/phy2.16057 (PMC11144550; doi:10.14814/phy2.16057)
Supplement: Supplementary file 3 — Table S1. [file PHY2-12-e16057-s001.zip › PHYSREP-2024-03-139-file003.docx]

**Supplemental material**

**Abbreviation:**

- LO-1 (Lung organoid culture medium-1)
- LO-2 (Lung organoid culture medium-2)
- EM (Expansion medium)
- DM (Differentiation medium)

**Supplemental table 1.** The culture medium components

|  | **Groups** | **LO-1** | **LO-2** | **EM** | **DM** | |
| --- | --- | --- | --- | --- | --- | --- |
|  | Wnt3a | 100ng/mL | 100ng/mL | / | / | |
|  | R-Spondin1 | 100ng/mL | 100ng/mL | 100ng/mL | 100ng/mL |  |
|  | B27 | 1X | 1X | 1X | 1X | |
|  | N-acetylcysteine | 1.25mM | 1mM | 1.25mM | 1.25mM | |
|  | Nicotinamide | 10mM | 10mM | 10mM | 10mM | |
|  | Noggin | 100ng/mL | 100ng/mL | 100ng/mL | 100ng/mL | |
|  | Gastrin I | 10Nm | / | / | / | |
|  | A83-01 | 0.5μM | 0.5μM | 0.5μM | 0.5μM | |
|  | Y-27632 | 10μM | 10μM | 10μM | 10μM | |
|  | HGF | 25ng/mL | / | / | / | |
|  | FGF-10 | 100ng/mL | / | 50ng/mL | 50ng/mL | |
|  | FGF-7 | / | 10ng/mL | 5ng/mL | 5ng/mL | |
|  | EGF | 50ng/mL | 100ng/mL | 50ng/mL | 50ng/mL | |
|  | CHIR99021 | / | 3 μM | 3 μM | 3 μM | |
|  | All-Trans Retinoic Acid | / | 50nM | 50nM | 50nM | |
|  | IBMX | / | / | / | 0.1mM | |
|  | 8-bromo-cAMP | / | / | / | 0.1mM | |
|  | Dexamethasone | / | / | / | 50nM | |

**Supplemental table 2.** Antibody resources table

| **Reagent type (species) or resource** | **Designation** | **Source or reference** | **Identifiers** | **Additional information** |
| --- | --- | --- | --- | --- |
| Antibody | cytockeratin 7 (CK7) | Abcam | ab154334 | IF 1/100 |
| Antibody | P63 | Abcam | ab124762 | IF 1/300 |
| Antibody | MUC5AC | Abcam | ab3649 | IF 1-2μg/ml |
| Antibody | SOX9 | Abcam | ab185966 | IF 1/200 |
| Antibody | AQP5 | SANTA CRUZ BIOTECHNOLOGY | sc-514022 | IF 1/200 |
| Antibody | SFTPC | Abcam | ab211326 | IF 1/500 |
| Antibody | cytokeratin 5(CK5) | Abcam | ab52635 | IF 1/100 |
| Antibody | Sodium Potassium ATPase | Abcam | ab76020 | IF 1/500 |
| Antibody | Goat anti-Mouse IgG | Invitrogen | #2465113 | IF 1/1000 |
| Antibody | Goat anti-Rabbit IgG | Invitrogen | #2379475 | IF 1/1000 |

**Supplemental table 3.** Sequences of primers for quantitative PCR

| **Gene** | **Forward primer** | **Reverse primer** |
| --- | --- | --- |
| ß-actin | 5’-GGCCAACCGTGAAAAGATGA-3’ | 5’-CAGCCTGGATGGCTAGTACA-3’ |
| Hopx | 5’-ATACTGTCCCCTCGGAGTGTC-3’ | 5’-GTGCGCGTCTGACTAAGGAT-3’ |
| PDPN | 5’-TTGTGACCCCAGGTACAGGA-3’ | 5’-TGGCAAGCCATCTCTATTGGG-3’ |
| AQP5 | 5’-TCTCTCCCGGAAGAATCGGA-3’ | 5’-TCTCTCCCGGAAGAATCGGA-3’ |
| Sftpc | 5’-GCACCGGAAACTCAGAAACG-3’ | 5’-TCTCTCCCGGAAGAATCGGA-3’ |
| NKX2.1 | 5’-AGGCAGTCGTTCCCTTACTC-3’ | 5’-TCATCGACATGATTCGGCGT-3’ |
